# Reversible Akinetic Mutism after Aneurysmal Subarachnoid Haemorrhage in the Territory of the Anterior Cerebral Artery without Permanent Ischaemic Damage to Anterior Cingulate Gyri

**DOI:** 10.1155/2016/5193825

**Published:** 2016-06-23

**Authors:** François-Xavier Sibille, Philippe Hantson, Thierry Duprez, Vincent van Pesch, Simone Giglioli

**Affiliations:** ^1^Department of Intensive Care, Université Catholique de Louvain, Cliniques St-Luc, 1200 Brussels, Belgium; ^2^Department of Neuroradiology, Université Catholique de Louvain, Cliniques St-Luc, 1200 Brussels, Belgium; ^3^Laboratory of Neurophysiology, Université Catholique de Louvain, Cliniques St-Luc, 1200 Brussels, Belgium

## Abstract

We report on two cases of transient akinetic mutism after massive subarachnoid haemorrhage due to the rupture of an intracranial aneurysm of the anterior cerebral artery (ACA). In the two cases, vasospasm could not be demonstrated by imaging studies throughout the clinical course. Both patients shared common radiological features: a hydrocephalus due to haemorrhagic contamination of the ventricular system and a mass effect of a subpial hematoma on the borders of the corpus callosum. Patients were also investigated using auditory event-related evoked potentials at acute stage. In contrast to previous observations of akinetic mutism, P300 wave could not be recorded. Both patients had good recovery and we hypothesized that this unexpectedly favourable outcome was due to the absence of permanent structural damage to the ACA territory, with only transient dysfunction due to a reversible mass effect on cingulate gyri.

## 1. Introduction

In contrast to the high incidence of subarachnoid haemorrhage (SAH) due the rupture of an intracranial aneurysm (RIA) of the anterior cerebral artery (ACA), akinetic mutism complicating vasospasm-related bilateral stroke within anterior cingulate gyri and supplementary motor area (SMA) remains uncommon [1–3]. We describe two cases of akinetic mutism occurring in the course of massive SAH from RIA in the ACA territory in whom diffusion-weighted MR imaging (DW-MRI) failed to reveal ischaemic damage to the cingulate gyri. Both patients experienced a rapid recovery. We speculate that mass effect synergistically due to oedematous changes, hydrocephalus, and subpial hematoma impinging on the corpus callosum may have led to reversible dysfunction of the cingulate gyri in the absence of permanent ischaemic damage. Both patients were also investigated at the acute phase using event-related auditory evoked potentials.

## 2. Case 1 Presentation

**Figure 1:**
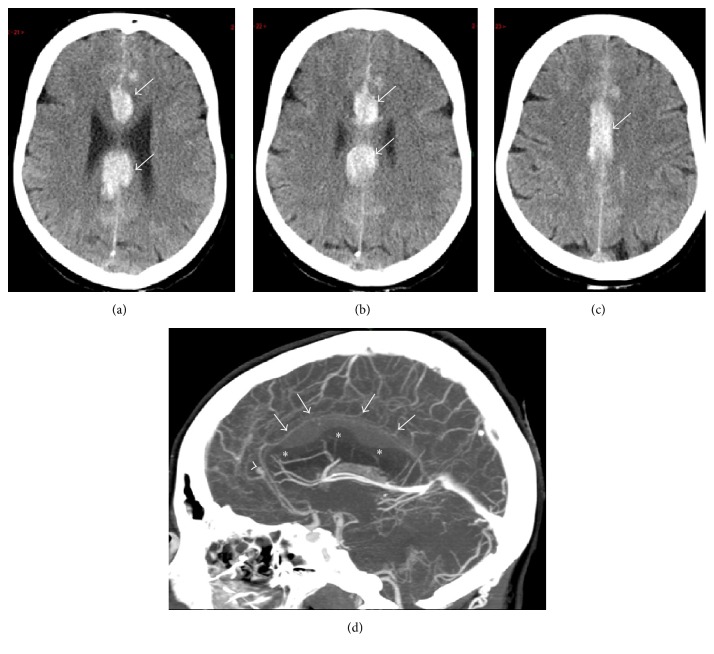
Admission brain CT scanner work-up of patient 1. (a–c) Serial unenhanced axial transverse views through the level of the corpus callosum showing massive hyperintense subpial hematoma at the cranial border of the corpus together (white arrows) with a mild SAH at the lateral borders of the frontal and parietal lobes, mainly on the left side. (d) Contrast-enhanced mid-sagittal reformatted Maximum Intensity Proton (MIP) view showing severe polylobulated impingement of the subpial hematoma (white arrows) on the corpus callosum (asterisks) and the causative microaneurysm on the left ACA (arrowhead). Note artifactual lowering of the apparent density of the hematoma because of changes in image scaling (window/level) after contrast agent injection.

**Figure 2:**
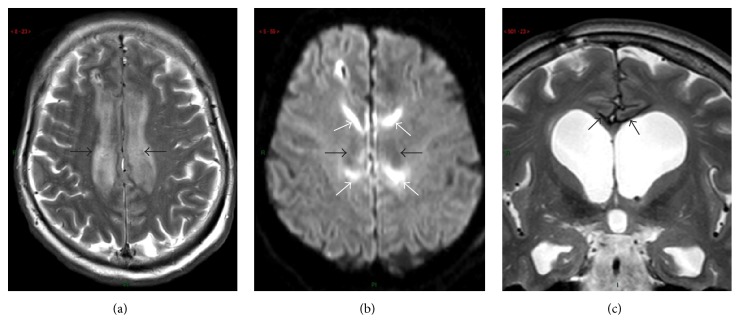
MRI work-up (a-b) at acute phase and delayed (7 months) MRI follow-up examination (c) of patient 1. (a) Axial transverse T2-weighted fast-spin echo (FSE) view through cingulate gyri demonstrating similar findings as in previous patient: oedema-related strong hypersignal intensity within them on both sides (black arrows). (b) Axial transverse diffusion-weighted (*b* factor = 1000 s/mm^2^) view in similar slice location (black arrows) failed to reveal hypersignal intensity, thereby excluding ischaemic cytotoxic damage. Only false positive artifacts due to adjacent hematoma were seen (white arrows). (c) Coronal T2-weighted FSE MR view at chronic phase (7 months) showed intrinsic textural integrity of the cingulate gyri. Meningeal hemosiderosis surrounding cingulate gyri featured by pial strong hyposignal intensity was present (arrows).

A 61-year-old woman with a heavy medical history of arterial hypertension, sarcoidosis, hypothyroidism, and bilateral optic nerve atrophy was referred to the Emergency Department for severe headache, vomiting, and alteration of consciousness. Her Glasgow Coma Score on admission was 15/15 but the patient had a slurry speech and exhibited choreic movements of the upper limbs. The admission brain computed tomography (CT scanner) revealed a Fisher grade 4 SAH with initially mild intraventricular bleeding uncomplicated by hydrocephalus. Angiographic examination confirmed the presence of a saccular aneurysm at the junction of the left pericallosal and callosomarginal arteries, which was successfully treated by coiling. The neurological status worsened a few hours after the procedure and an early follow-up brain CT scanner was performed with increased hydrocephalus and the* de novo* constitution of a subpial hematoma collected at the upper border of the corpus callosum (Figure 1). Extubation was possible soon after surgery for intraventricular drainage. The neurological examination showed spontaneous eye opening and some spontaneous movements of the upper and lower limbs with a mild left hemiparesia. When stimulated, the patient remained completely mutic but demonstrated some orientating reactions toward the source of intense verbal and/or auditory stimulation. A brain magnetic resonance imaging (MRI) performed on day 6 failed to reveal vasospasm or ischaemic injury in both ACA territories (Figures 2(a)-2(b)). In contrast, there was a significant impingement by the mass effect of the subpial hematoma on the superior border of the corpus callosum. Expectedly, major haemorrhagic contamination of the whole ventricular system was still present.

Event-related evoked potentials were recorded on days 13 and 28, using an auditory oddball paradigm (at least two independent acquisitions of 100 stimuli, consisting of an 85 dBHL binaural tone burst at 500 Hz for the frequent stimulus and 750 Hz for the rare stimulus, with a standard/deviant ratio of 85/15 and an interstimulus interval of 0,8 Hz). Exogenous activities were normal (N100: 85 ms; P200: 120 ms). In contrast, no endogenous activities were obtained.

The patient was discharged from the Intensive Care Unit on day 11 with persisting mutism and some uncontrolled movements in the right hemibody and left hemiparesia. Levodopa (125 mg orally t.i.d) had been initiated from day 7 but was stopped at the time of her transfer to the rehabilitation clinic on day 27. At this moment, the patient was still mutic and unable to produce spontaneous movements. Mutism almost resolved after 6 weeks. At 6-month follow-up, the patient had a fluid speech with minor episodes of dysarthria. The recovery of neurocognitive functions was very satisfactory, with a mild fatigability impairing attention demanding tasks. Memory was well preserved. Slower improvement was noted regarding motor recovery. After 6 months, the patient was able to walk with some help and could return home to follow an outpatient rehabilitation program. At 7-month follow-up, brain MRI was repeated and documented the integrity of cingulate gyri (Figure 2(c)). At 12-month follow-up, some postural instability persisted but language had completely recovered.

## 3. Case 2 Presentation

**Figure 3:**
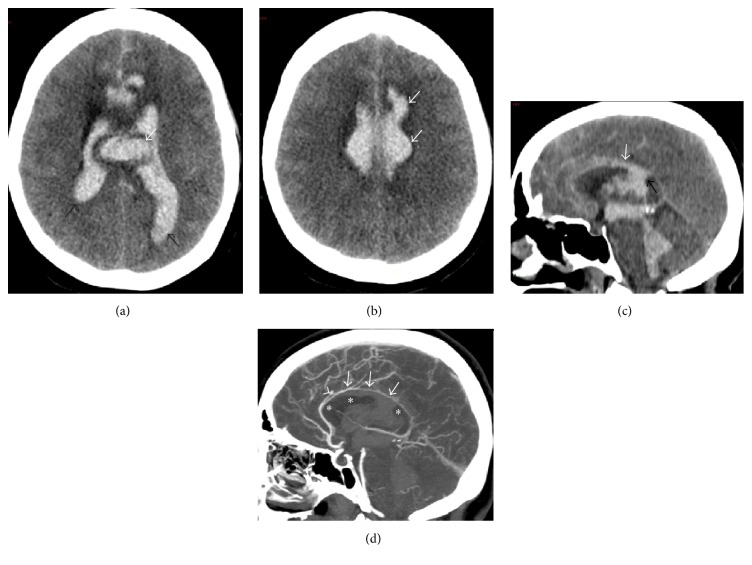
Admission CT scanner work-up of patient 2. (a) Unenhanced axial transverse view through the corpus callosum showing ovoid-shaped hematoma within the mid-posterior area of the body of the corpus (white arrow), anterior interfrontal SAH, and massive haemorrhagic contamination of the whole ventricular system (black arrows). (b) Unenhanced axial transverse view tangent through the cranial aspect of the corpus callosum showing subpial fresh hematoma (arrows). (c) Unenhanced mid-sagittal reformatted view showing filling of the ventricular system by acute hyperintense blood, transgression of the posterior part of the body of the corpus callosum by fresh blood (black arrow), and subpial fresh hematoma at the upper border of the corpus callosum (white arrow). (d) Contrast-enhanced mid-sagittal reformatted view showing the causative RIA of the left ACA (arrowhead) and the subpial hematoma (arrows) on the cranial aspect of the corpus callosum (asterisks). Again, artifactual lowering of the apparent density of the hematoma because of changes in image scaling (window/level) after contrast agent injection (similarly as seen on Figure 1(d)).

**Figure 4:**
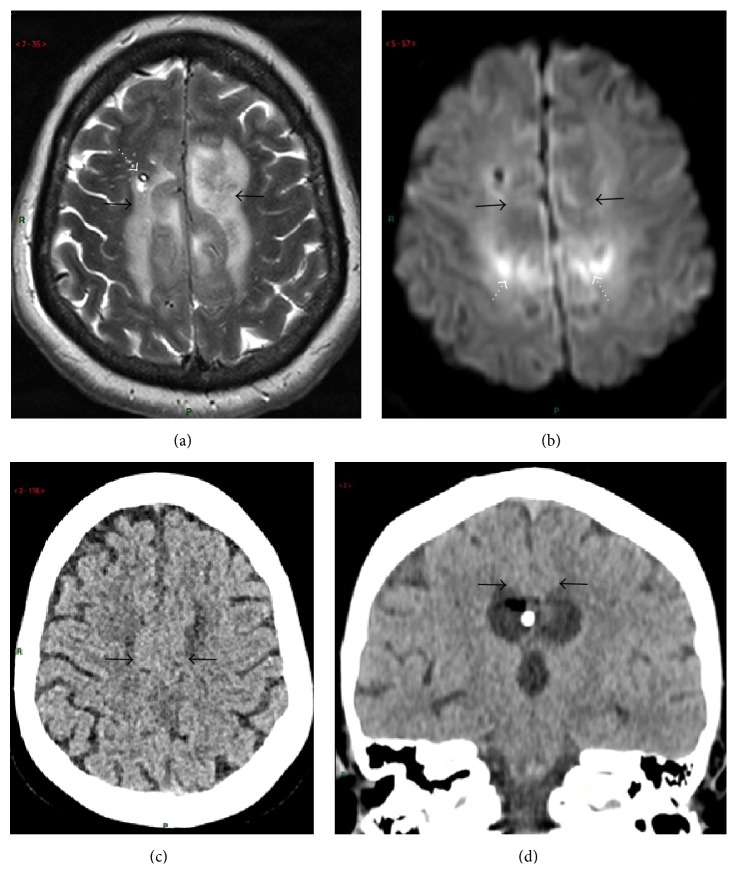
MRI work-up (a-b) at acute phase and delayed (4 months) CT scanner follow-up imaging (c-d) of patient 2. (a) T2-weighted fast-spin echo (FSE) axial transverse view through cingulate gyri demonstrating oedema-related strong hypersignal intensity within them on both sides. Ventricular draining catheter is seen on right frontal area (dotted white arrow). (b) Axial transverse diffusion-weighted (*b* factor = 1000 s/mm^2^) view in similar slice location failed to reveal hypersignal intensity within the same cingular areas (black arrows), thereby excluding ischaemic cytotoxic oedema. Only false positive artifacts due to adjacent hematoma were seen (arrows). (c-d) CT scanner axial transverse (c) and coronal reformatted (d) images from helical acquisition demonstrating integrity of the cingulate gyri (arrows).

A 56-year-old woman was found unconscious at home and transferred to the Emergency Department with a Glasgow Coma Score of 5/15 (E2V1 M2). The admission brain CT scanner revealed the presence of a Fisher grade 4 subarachnoid haemorrhage. Secondary hypertensive hydrocephalus was present together with a subpial dissecting hematoma at the posterior part of the corpus callosum (Figure 3). Angiographic examination confirmed the presence of a saccular microaneurysm arising at the junction of the right pericallosal and callosomarginal arteries. An external ventricular drain was immediately placed and her neurological status improved subsequently. The aneurysm was successfully treated by coiling. On day 10, the patient had spontaneous eye opening and weak motor responses to nociceptive stimuli. Verbal response could not be evaluated as the patient was still on mechanical ventilation. The brain MRI (day 7) showed a complete haemorrhagic necrosis of the corpus callosum with a hematoma transfixing the posterior part of the body of the corpus and a bilateral oedema of the cingulate gyri (Figures 4(a)-4(b)). The ventricular system was completely filled with blood in spite of ventricular drainage. No vasospasm or secondary ischaemic lesions could be demonstrated. After the complete weaning from the mechanical ventilation and removal of the tracheostomy (day 22), the patient seemed to understand verbal commands. She was totally mutic and tetraparetic but initiated some movements of the head and neck for “yes” or “no.”

Levodopa therapy (125 mg orally t.i.d) was started on day 29 and interrupted on day 48.

The follow-up brain CT scanner after 4 months also demonstrated the integrity of cingulate gyri (Figures 4(c)-4(d)).

Event-related evoked potentials had been performed at the acute phase (day 9) using an oddball auditory paradigm. Only exogenous activities were obtained but were delayed (N100: 170 ms; P200: 250 ms). There were no endogenous activities. The patient was transferred to the rehabilitation ward after one month with persisting akinetic mutism. At 6-month follow-up, the patient had fully recovered speech fluency. She suffered, however, from anxiety and apraxia which was likely related to callosal injury. She was unable to return to work and was transferred to a nursing home. She had regained autonomy for daily life activities but still required some help for postural changes.

A control of brain MRI was planned at 6 months, but the examination had to be interrupted due to uncontrollable anxiety and claustrophobia. We failed to repeat evoked potentials recording for the same reasons. At 12-month follow-up, her condition is relatively unchanged and a new neurocognitive testing is planned.

## 4. Discussion 

The term of “akinetic mutism” (AM) was first introduced by Cairns and coworkers in 1941 to describe a syndrome featured by a lack of responsiveness in spite of apparently preserved vigilance [4]. Clinical examination usually reveals a complete or almost complete immobility and a lack of verbal contact [2]. Anatomically, different brain structures may be involved, with a possible distinction between a telencephalic form (affecting medial frontal lobes, cingulate gyri), a diencephalic form (thalamic/basal ganglia), and a mesencephalic form (upper brainstem reticular activating system). AM is usually observed in the frame of head trauma, brain neoplasia, or ischaemic stroke, but rarely after aneurysmal subarachnoid haemorrhage, and particularly as a presenting feature [1]. A comprehensive literature review performed by Choudhari in 2004 retrieved only a total of 21 published cases of this rare condition [1, 2, 5–15]. Most of the patients usually presented with secondary bilateral infarction in the ACA territory and had poor prognosis. Bilateral damage to anterior cingulate gyri and supplementary motor area, which results in most cases from ischaemic injury in the territories of both ACAs, is often associated with the syndrome. As for hydrocephalus, it has also been implicated in a delayed form of AM following SAH [16]. Only a single previous case of AM without structural lesions, but with bilateral frontal lobe dysfunction demonstrated by single-photon emission computed tomography (SPECT), has been described [5].

Among the neurophysiological tools that can be performed at the bedside, auditory cognitive event-related potentials (ERPs) were rarely explored in patients presenting with AM. In a 38-year-old woman with severe AM due to large bilateral ACA infarction due to RIA-related vasospasm, Naccache et al. recorded a “Mismatch Negativity” (MMN) and a larger P300 wave in rare trials than in frequent ones using the passive auditory oddball paradigm [6]. These findings may reflect the persistence of a high level of cognitive integration of current environmental stimuli in case of severe AM and confirmed a similar previous observation [7]. The MMN reflects an automatic preattentive processing stage that seems to be generated in the temporal lobe [8]; the P300 wave is a more complex integrative processing that involves both cortical and subcortical networks [9]. The neural networks involved in the generation of the P300a component are located not only within the frontal cortex and anterior cingulate gyri but also in parietal, temporal, and occipital regions. The generators of the P300b component originate from the posterior part of the cingulate gyri and also from parietal and temporal lobes [10, 11].

The relevance of MMN and P300 recordings in AM has still not been assessed. One may suggest it could reflect the preservation of a high level of cognitive integration at the acute stage of AM despite poor common neurological testing.

In both cases reported here, no auditory ERPs could be elicited, in contrast to previous reports and despite absence of ischaemic damage of the anterior cingulate gyri and the supplementary motor areas. This is not totally explained by callosal injury or dysfunction, as some authors have documented the preservation of ERPs in patients with callosal disconnection [12, 13]. It could be due to the early timing of the ERP recording but an alternative hypothesis could be that the cortico-subcortical dysfunction involved areas larger than the frontal lobes, thus preventing generation of the P300 components [12, 13]. The discrepancy between the documentation of some orienting reactions to noise or verbal stimuli in both cases and the absence of P300 can be explained by the fact that the ERP stimulus is a pure beep tone in contrast to a richer and more complex auditory stimulus coming from the environment.

The prognosis of AM following SAH is usually poor, expectedly in case of permanent damage to cingulate gyri. In turn, the prognosis appears better when AM is due to hydrocephalus or reversible compression of cingulate gyri similarly to both our patients. There is no existing data that surgical removal of the hematoma could impact on the clinical course. Data regarding the systematic use of dopamine agonists for patients with akinetic mutism are inconclusive. There have been reports that treatment with dopaminergic drugs is useful in akinetic mutism due to lesions at the diencephalon [14]. Dopaminergic agents should be less effective if the lesion is involving the anterior cingulate cortex because dopaminergic receptors would have been destroyed [15]. In our two patients, levodopa withdrawal did not result in clinical deterioration.
